# Prediction of 24-h and 6-h Periods before Calving Using a Multimodal Tail-Attached Device Equipped with a Thermistor and 3-Axis Accelerometer through Supervised Machine Learning

**DOI:** 10.3390/ani12162095

**Published:** 2022-08-16

**Authors:** Shogo Higaki, Yoshitaka Matsui, Yosuke Sasaki, Keiko Takahashi, Kazuyuki Honkawa, Yoichiro Horii, Tomoya Minamino, Tomoko Suda, Koji Yoshioka

**Affiliations:** 1 National Institute of Animal Health, National Agriculture and Food Research Organization, Tsukuba 305-0856, Japan; 2 Dairy Cattle Group, Dairy Research Center, Hokkaido Research Organization, Nakashibetsu 086-1135, Japan; 3 Department of Agriculture, School of Agriculture, Meiji University, Kawasaki 214-8571, Japan; 4 Department of Industry and Economy, Agricultural Technology Promotion Division, Okitama General Branch Office, Yamagata Prefecture Government, Takahata 999-2174, Japan; 5 Division of Research and Training for Livestock and Veterinary Clinic, Honkawa Ranch, Hita 877-0056, Japan; 6 Laboratory of Theriogenology, School of Veterinary Medicine, Azabu University, Sagamihara 252-5201, Japan

**Keywords:** acceleration, cow, parturition prediction, skin temperature, support vector machine, wearable device

## Abstract

**Simple Summary:**

Routine visual observation of signs of imminent calving, such as softening of ligaments around the tailhead and udder distension, is time-consuming, and the resulting calving predictions are relatively unreliable. To address this issue, we used a multimodal tail-attached device (tail sensor) and developed calving prediction models through supervised machine learning. The tail sensor is equipped with a thermistor and 3-axis accelerometer, and can monitor tail skin temperature, activity intensity, lying time, posture changes (standing to lying or vice versa), and tail raising behavior. Using the sensor data with a non-sensor-based data (days to the expected calving date), we developed calving prediction models for 24-h and 6-h periods before calving and evaluated their predictive ability under two distinct housing conditions, tethering (tie-stall) and untethering (free-stall and individual pen). Our results demonstrated that calving prediction models based on tail sensor data with supervised machine learning have the potential to achieve effective calving prediction, irrespective of the cattle housing conditions.

**Abstract:**

In this study, we developed calving prediction models for 24-h and 6-h periods before calving using data on physiological (tail skin temperature) and behavioral (activity intensity, lying time, posture change, and tail raising) parameters obtained using a multimodal tail-attached device (tail sensor). The efficiencies of the models were validated under tethering (tie-stall) and untethering (free-stall and individual pen) conditions. Data were collected from 33 and 30 pregnant cattle under tethering and untethering conditions, respectively, from approximately 15 days before the expected calving date. Based on pre-calving changes, 40 features (8 physiological and 32 behavioral) were extracted from the sensor data, and one non-sensor-based feature (days to the expected calving date) was added to develop models using a support vector machine. Cross-validation showed that calving within the next 24 h under tethering and untethering conditions was predicted with a sensitivity of 97% and 93% and precision of 80% and 76%, respectively, while calving within the next 6 h was predicted with a sensitivity of 91% and 90% and precision of 88% and 90%, respectively. Calving prediction models based on the tail sensor data with supervised machine learning have the potential to achieve effective calving prediction, irrespective of the cattle housing conditions.

## 1. Introduction

The advantages of precise calving prediction include increasing the possibility of attendance at calving and reducing unnecessary supervision of pregnant cattle. Timely and appropriate calving assistance is associated with a significant decrease in the mortality of calves and dams [1,2] and improved reproductive performance during the subsequent lactation period [3,4]. However, routine visual observation of pre-calving clinical signs, such as relaxation of the pelvic ligament and udder distension, is time-consuming, and the resulting predictions are relatively unreliable. Sensitivity (the proportion of calving correctly predicted among total calving events) and precision (the proportion of calving correctly predicted among total calving predicted) were reported to be 89% and 15%, respectively, in predicting calving within a 12 h period [5]. Therefore, numerous studies have been conducted on automatic and objective calving prediction using wearable devices [6].

Many physiological and behavioral changes have been identified as predictors of calving using wearable devices [7]. Regarding physiological changes, pre-calving de-creases in body temperature can be detected by monitoring vaginal, reticuloruminal, or body surface temperature and calving can be predicted within a 24 h period with a sensi-tivity of 44–86% and a precision of 37–80% [8,9,10,11,12]. Regarding behavioral changes, such as decreasing lying time, increasing lying bouts, decreasing rumination time, and a combi-nation of these changes allows the prediction of calving within a 24 h period with a sensi-tivity of 54–75% and a precision of 29–34% [11]. Recently, tail-mounted inclinometer sen-sors have been used to monitor calving-associated tail-raising behavior, and there is a growing consensus that tail raising is one of the most consistent behavioral changes ob-served in the hours before calving [13]. However, the prediction performance based on the tail-raising behavior varied greatly depending on the prediction period preceding the onset of calving; sensitivities and precisions were 19–75% and 9–65%, respectively, when calving next 1, 2, 4, 12, and 24 h were predicted [14].

Both the above-mentioned physiological and behavioral parameters change during the pre-calving period; thus, combining these parameters is hypothesized to mitigate individual drawbacks (i.e., false positive and false negative predictions) and maximize predictive performance. However, to the best of our knowledge, only one study has used single-device monitoring of both parameters [15]. In the study, calving prediction models were developed based on the data obtained by a commercially available ear-attached device (Agis SensOor, Agis Automatisering B.V., Harmelen, The Nether-lands), which measures ear temperature, activity level, rumination time, and feeding time. Calving within the next 24 h and 6 h was predicted with a sensitivity of 36% and 49% and a precision of 60% and 11%, respectively [15]. Owing to the relatively low predictive performance of the models, the authors suggested adding behavioral or physiological variables more directly related to calving for more specific prediction, such as the data on standing and lying patterns [15].

In the present study, we used a multimodal tail-attached device (tail sensor) that can monitor both physiological (tail skin temperature: ST) and behavioral (activity intensity, lying time, posture changes: standing to lying or vice versa, and tail raising) parameters. We developed two calving prediction models for predicting the 24-h and 6-h periods before calving. This is because these two-stage calving alerts might be helpful in timely checking signs of the first- and second-stage of calving for proper calving management [16]. Furthermore, calving prediction models were developed for tethering and untethering conditions, and the predictive ability of the models was evaluated under the respective conditions. Because, although verification in tethered cattle is necessary for practical use since significant cattle herds are housed in tethering conditions (tie-stall systems) in many countries [17], most previous studies have focused only on untethered cattle. The objective of this study was to determine the effectiveness of calving prediction models based on the tail sensor data with supervised machine learning under two distinct housing conditions, tethering and untethering. We hypothesized that the calving prediction models might produce appropriate two-stage calving alerts useful for ensuring proper calving management, irrespective of the cattle housing conditions.

## 2. Materials and Methods

### 2.1. Animals

Experiments were conducted under two housing systems, tethering (n = 33) and un-tethering (n = 30), from June 2019 to February 2021. A total of 63 healthy pregnant cattle (1.8 ± 1.8 parities) were enrolled in this study. In the tethering system, 33 pregnant Hol-stein cattle conceived single Holstein fetuses and were housed in a sawdust-bedded tie-stall barn. In the untethering system, 9 Holstein cattle (2, 2, and 5 cattle conceived Hol-stein, Japanese Black, and Holstein–Japanese Black crossbred fetuses, respectively), 3 Brown Swiss cattle (all cattle conceived Brown Swiss fetuses), and 18 Japanese Black cattle (all cattle conceived Japanese Black fetuses) were housed in sawdust-bedded free-stall barns or individual pens. All cattle were reared under natural temperature conditions (daily average ambient temperature under the tethering and untethering housing conditions were 17.4 ± 4.4 °C and 18.6 ± 8.5 °C, respectively, during the experimental period) and fed rations to meet the Japanese Feeding Standard recommendations, with ad libitum access to water.

Pregnancy was confirmed via transrectal ultrasonography approximately 30 and 60 days post-insemination. The expected calving date was calculated from the breeding rec-ords, assuming 280, 290, 290, and 285 days for Holstein, Brown Swiss, Japanese Black, and Holstein–Japanese Black crossbred fetuses, respectively [18,19]. Pregnant cattle were moved to an individual maternity pen (3–6 m × 3–6 m) when they showed premonitory signs of calving (i.e., restlessness, pelvic ligament relaxation, and teat filling). Calving as-sistance was provided to the cattle when the farm staff detected abnormalities in calving progress. The cattle were continuously monitored for calving through video recording or visual observation by farm staff, and the calving time, defined as the time when the fetus was expelled from the vulva, was recorded.

### 2.2. Multimodal Tail-Attached Device (Tail Sensor) and Sensor Data Collection

**Figure 1:**
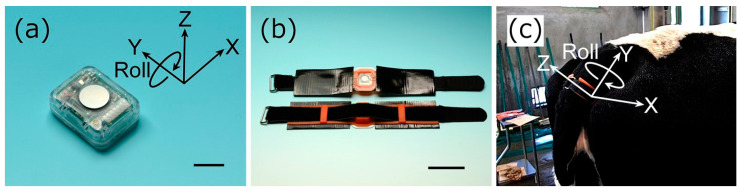
External appearance and usage of the multimodal tail-attached device (tail sensor). (**a**) External appearance of a tail sensor designed for fitting the ventral tail base of cattle. The sensor was attached so that the stainless steel plate for temperature measurement (the silver round part) was on the animal side. X, Y, and Z denote the axes of the 3-axis accelerometer. Roll indicates the rotation of the X- and Z-axes about the Y-axis. The sensor dimensions are 21.0 mm × 26.0 mm × 9.7 mm, and the sensor weight with the battery is 5.8 g. (**b**) Tail sensor in an attachment. The sensor was inserted into a pocket formed in a silicone rubber belt (orange part) and sealed with a urethane gel sheet. (**c**) Position of the tail sensor. The silicone rubber belt with the sensor was attached to the ventral tail base using double-sided adhesive tape, and its position was stabilized using a hook-and-loop fastener. The orientations of the X-, Y-, and Z-axes were lateral, proximal/distal, and dorsal/ventral to the tail, respectively. The bars in (**a**,**b**) are 1 cm and 5 cm, respectively.

In the present study, we used a tail sensor that can monitor both physiological (ST) and behavioral (activity intensity, lying time, posture changes, and tail raising) parameters. The tail sensor was designed to fit the ventral tail base of cattle, as previously described (Figure 1a) [20]. The sensor comprises a thermistor and 3-axis accelerometer, and its dimensions are 21.0 mm × 26.0 mm × 9.7 mm, weighing 5.8 g with the battery. The sensor was fitted using a dedicated attachment, as described in a previous study (Figure 1b) [10]. Briefly, the sensor was inserted into a pocket formed in a silicone rubber belt and sealed with a urethane gel sheet. The silicone rubber belt with the sensor was then attached to the ventral tail base using double-sided adhesive tape, and its position was stabilized using a hook-and-loop fastener. Orientations of the X-, Y-, and Z-axes of the 3-axis accelerometer were lateral, proximal/distal, and dorsal/ventral to the tail, respectively (Figure 1c).

The tail sensor measured ST (in the range of 20 to 45 °C, with 0.05 °C resolution), ac-tivity intensity (in the range of 0 to 102.3, with 0.2 resolution), roll angle (rotation of the X- and Z-axes about the Y-axis: range of −3 to +3 rad, with 0.05-rad resolution), and Y-axis acceleration (in the range of −1000 to +1000 mg, with 4 mg resolution). The Y-axis acceler-ation has positive, zero, and negative values when the tail is down, horizontal, and raised above the horizontal, respectively. Therefore, hereinafter, Y-axis acceleration is referred as “tail raising index”.

The sensor was attached to the pregnant cattle from 15.0 ± 4.6 days (n = 63) before the expected calving date until at least one day after calving. The sensor wirelessly transmitted the data to the receiver in real-time at 3-min intervals. The carrier frequency of the transceiver was 920 MHz, and the communication distance to the receiver was approximately 100 m without obstacles. The data were uploaded to the cloud server via the Global System for Mobile Communication. The data were downloaded from the cloud server as comma-separated value files for the analysis.

### 2.3. Data Preprocessing and Feature Extraction

The maximum hourly ST, hourly averaged activity intensity, and hourly averaged tail raising index were calculated to minimize the impact of rapid momentary changes in the raw data. Based on the roll angle, the posture of the animals (standing or lying) was determined at each time point (3-min interval) [20], and the hourly lying time and the number of posture changes were calculated.

After cleaning the data for missing values by forward-filling (i.e., taking the last known value and using this to fill), ST changes were expressed as residual ST (rST = actu-al hourly ST − mean ST for the same hour on the previous three days) to exclude the in-fluence of the circadian rhythm [21]. Similarly, activity intensity and lying time were ex-pressed as the activity ratio and lying time ratio, respectively, calculated as the total value during the last 24 h/total value during the last 25–48 h. In the posture change and tail-raising behavior, there were no evident circadian rhythms and their pre-calving changes occur in a relatively short time window; posture change becomes more frequent from approximately 6 to 12 h before calving [11,22,23,24] and tail-raising behavior increases from approximately 2 to 6 h before calving [22,25]. Therefore, to detect these changes, the posture change ratio was calculated as the average number during the last 6 h/average number during the last 7–30 h, and the tail raising index ratio was calculated as the average value during the last 3 h/average value during the last 4–27 h.

In the rST data, there were many short-term (within a few hours) small ups and downs; therefore, an exponentially weighted moving average (EWMA) was applied to smoothen the data. The EWMA was calculated using the following formula:

EWMA_0_ = X_0_

EWMA_t_ = αX_t_ + (1 − α)EWMA_(t−1)_, t > 0,

where EWMA_t_ is the EWMA at time t, X_t_ is the measured value at time t, and EWMA_t−1_ is EWMA at time t − 1. Parameter α, an adjustable smoothing parameter with a value between 0 and 1, was set to 0.1 based on a previous study [26].

Without monitoring its sequential changes, a given numerical value is not necessari-ly indicative of calving prediction. Therefore, a total of 40 features (8 physiological and 32 behavioral) that could reflect pre-calving physiological and behavioral changes were ex-tracted from the sensor data following previous studies [10,15,20] (Table 1). We also used one non-sensor-based feature (days to the expected calving date) because most of the calvings occur around the expected calving date [18,19]. Only the features that could be calculated from the current and past data were used to ensure that the calving prediction models applied to actual situations.

### 2.4. Development and Validation of Calving Prediction Models

Two binary classification models, which can predict 24-h and 6-h periods before calving for a particular time point, were developed under tethering and untethering conditions for a total of four calving prediction models (two prediction periods × two housing conditions). The leave-out-one animal (LOOA) cross-validation method [27] was used to develop and evaluate calving prediction models. Each LOOA run involving data from one animal was considered the test set, and the combined data from the remaining animals were considered the training set. Each was a test animal; therefore, 33 and 30 iterations were performed in the tethering and untethering conditions, respectively.

To develop 24-h calving prediction models, the training set data from 24 h before to 6 h after calving were labeled as positive instances, and the remaining data were labeled as negative instances. To develop 6-h calving prediction models, the training set data from 6 h before to 6 h after calving were labeled as positive instances, and the remaining data were labeled as negative instances. These data were used to generate calving prediction models using a support vector machine with the package e1071 [28] of R version 3.4.0. Hyperparameters for the 24-h (tethering/untethering conditions; sigma = 0.008/0.0037 and cost = 0.46/0.026, with a positive class weight of 1.5/3.5) and 6-h (tethering/untethering conditions; sigma = 0.008/0.0037 and cost = 0.1/0.03, with a positive class weight of 1.5/3.5) prediction models were manually selected with reference to the results of grid search performed on the training data sets using the function “tune” from the package e1071. All features were scaled to zero mean and unit variance to avoid problems due to the different orders of magnitude of the data [28].

Following the development of the models, the unlabeled data of the test set were ap-plied to the models and classified into “positive” or “negative” every hour. In the 24-h prediction model, at least three consecutive hours of positive predictions were regarded as a 24-h calving alert to minimize excessive alerts. If two or more alerts were presented within 12 h, they were assumed to be related and counted as a single alert. In the 6-h pre-diction model, every single positive prediction that overlapped with the positives in the 24-h prediction model was regarded as a 6-h calving alert. These alerts were regarded as true positive predictors when calving occurred within 24 h of 24-h calving alerts or 6 h of 6-h calving alerts. Non-alerted calving events and alerted non-calving events were re-garded as false-negative and false-positive predictors, respectively. Sensitivity (true-positive rate) and precision (positive predictive value) were calculated as (true-positive/[true-positive + false-negative]) and (true-positive/[true-positive + false-positive]), respectively [29].

Feature importance was calculated using the permutation importance technique with the function “FeatureImp” from the package iml [30] of R on the models developed using all data sets. In this method, the importance is measured as the factor by which the model’s prediction error (or loss of performance) increases when the feature is shuffled (permuted) [30].

### 2.5. Statistical Analysis

The hourly values of rST and ratios of activity intensity, lying time, posture change, and tail raising index from −120 h to 24 h of calving were compared with the corresponding values during the reference period (−240 h to −121 h from calving) using the Steel test. The sensitivity and precision of the calving prediction models developed under different housing conditions were compared using Fisher’s exact test and generalized score statistic [31,32], respectively. The mean intervals from calving alerts to actual calving were compared using a two-tailed Student’s *t*-test. Statistical analyses were performed using R and KyPlot software (ver. 6.0; KyensLab Inc., Tokyo, Japan), and differences were considered significant when the *p*-value was < 0.05. Values are presented as the mean ± standard deviation unless otherwise specified.

## 3. Results

In tethering and untethering conditions, all 63 births were single, and none of the tested cattle developed severe dystocia (i.e., requiring surgery or fetotomy [33]). In tethering condition, the tested cattle were moved to an individual maternity pen at 14.7 ± 9.1 h before calving, and all cattle calved between seven days before and after the expected calving date, with a mean of −0.5 ± 2.9 days (n = 33). In untethering condition, 80.0% of the cattle calved between seven days before and after the expected calving date, with a mean of 1.4 ± 4.5 days (n = 30). Attaching the sensor did not cause any severe lesions in cattle, although temporary depilation was eventually observed in all tested cattle owing to the adhesive tape of the sensor attachment.

### 3.1. Changes in Physiological and Behavioral Variables around Calving

**Figure 2:**
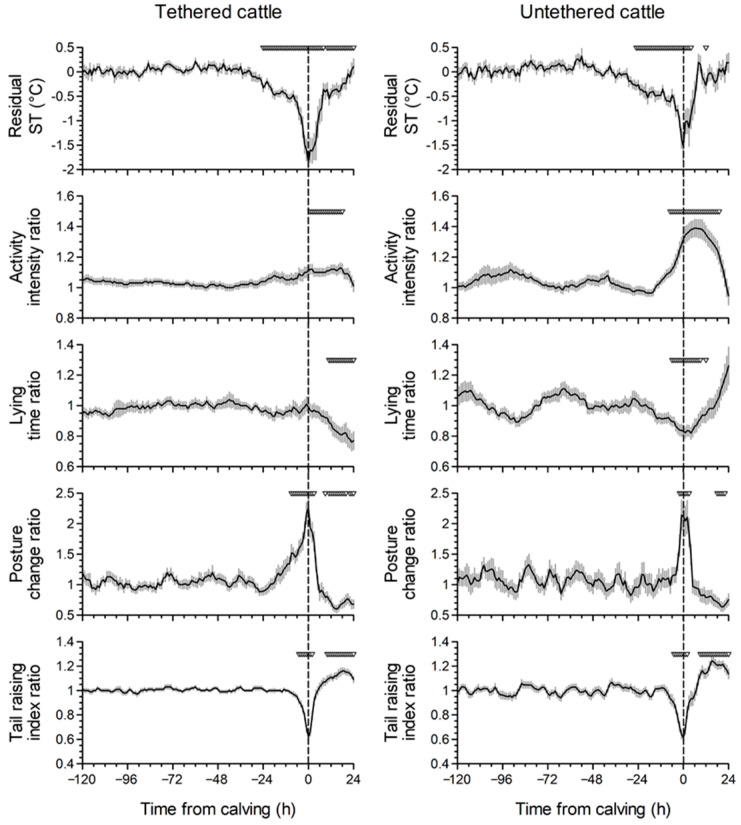
Changes in residual tail skin temperature (rST) and ratios of activity intensity, lying time, posture change, and tail raising index around calving. The rST was calculated as the actual ST − mean ST for the same time on the previous three days. The ratios of activity intensity and lying time were calculated as the total value during the last 24 h/total value during the last 25–48 h. The ratio of posture change was calculated as the average number during the last 6 h/average number during the last 7–30 h. The ratio of tail raising index was calculated as the average value during the last 3 h/average value during the last 4–27 h. Data were standardized to actual calving time (0 h: dashed vertical line). Inverted triangles indicate the time points with significant differences between the mean values at the indicated time points and the mean values during the reference period (−240 h to −121 h from calving) (*p* < 0.05). Data are expressed as the mean ± standard error (n = 33 and 30 in tethered and untethered cattle, respectively).

Figure 2 shows the changes in rST and the ratios of activity intensity, lying time, posture change, and tail raising index around calving. Regardless of the housing conditions, the mean rST decreased biphasically before calving: a gradual decrease from 36 to 16 h before calving and a subsequent sharp decrease during the last 6 h. In addition, the mean rST values during the last 24 h before calving were significantly lower than that during the reference period (−240 h to −121 h from calving) under both tethering and untethering conditions.

Before calving, the mean ratios of activity intensity and lying time were constant under tethering condition. However, under the untethering condition, the mean activity intensity ratio increased from approximately 18 h before calving, and the values during the last 7 h were higher than that during the reference period (*p* < 0.05). The mean lying time ratio decreased from approximately 24 h before calving in untethered cattle, and the values during the last 6 h were lower than that during the reference period (*p* < 0.05), while they did not change before calving under the tethering condition. The mean posture change ratio increased from approximately 24 h and 6 h before calving under tethering and untethering conditions, respectively, and these values during the last 9 h and 2 h were higher than the corresponding values during the reference period (*p* < 0.05). In both housing conditions, the mean tail raising index ratio decreased from approximately 12 h before calving, and the values during the last 5 h were lower than the value during the reference period (*p* < 0.05).

### 3.2. Calving Prediction Using Supervised Machine Learning

Table 2 shows the performance of the 24-h and 6-h calving prediction models for tethering and untethering conditions. The sensitivities and precisions of the models de-veloped under tethering and untethering conditions were not statistically different under the corresponding prediction periods. In total, calving within the 24 h and 6 h periods were predicted with a sensitivity of 95.2% and 90.5%, respectively, and a precision of 77.9% and 89.1%, respectively. The average time intervals from the true-positive calving alerts to the actual calving of the prediction models developed under the tethering and untethering conditions were also not statistically different under the corresponding pre-diction periods. In total, the mean intervals from 24-h and 6-h calving alerts to actual calving were 12.0 h and 2.5 h, respectively.

**Figure 3:**
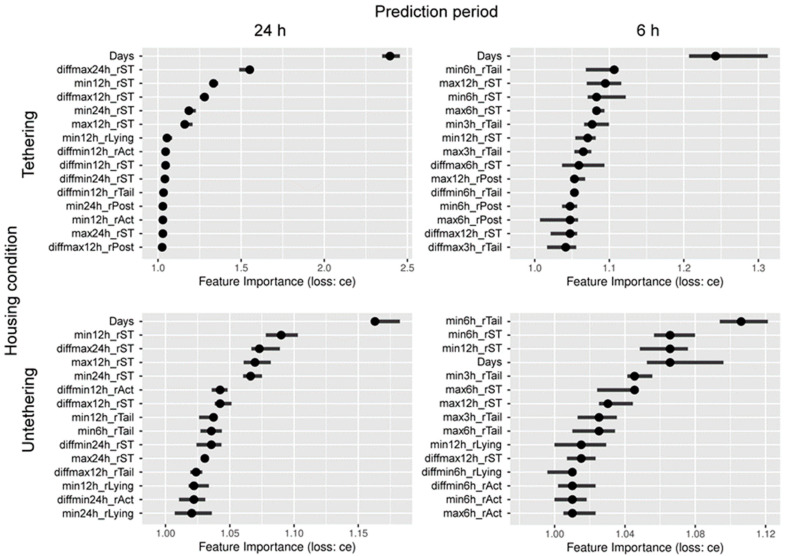
Permutation feature importance for predicting 24-h and 6-h periods before calving under tethering and untethering housing conditions (15 most important features out of 41 features used). Classification error loss function (loss: ce) was used to calculate the relative importance of each feature. Black circles denote median importance, and the horizontal lines denote the 90%-quantile of importance values. Abbreviations indicate as follows: Days, days to the expected calving date; minXh, minimum value during the last X h (X = 3, 6, 12, or 24); maxXh, maximum value during the last X h; diffminXh, difference between the current value and minimum value during the last X h; diffmaxXh, difference between the current value and maximum value during the last X h; rST, residual skin temperature; rAct, ratio of activity intensity; rLying, ratio of lying time; rPost, ratio of posture change; rTail, ratio of tail raising index.

Figure 3 shows the permutation feature importance for four calving prediction models (two prediction periods × two housing conditions). Regardless of the models, non-sensor-based feature “Days” (days to the expected calving date) was regarded as an important feature. Within the features derived from sensor data, the five or four most important features were rST-related for the models predicting 24-h period before calving under tethering and untethering conditions, while several behavioral parameters-related features (such as lying time- and activity intensity-related features) were also found to be more important than the rST-related features. For the models predicting 6-h period before calving, the feature “min6h_rTail” (minimum ratio of tail raising index during the last 6 h) was regarded as the most important sensor-based feature followed by rST-related features under both tethering and untethering conditions.

## 4. Discussion

In this study, we developed efficient calving prediction models for predicting 24-h and 6-h periods before calving using data on physiological (ST) and behavioral (activity intensity, lying time, posture change, and tail raising) parameters obtained by a tail sensor together with a non-sensor-based data “days to the expected calving date”. The efficiency of the calving prediction models was validated under two distinct housing conditions, tethering and untethering. To the best of our knowledge, this is the first report for the successful use of physiological and behavioral parameters for calving prediction through supervised machine learning in cattle.

The predictive performance of the present calving prediction models is higher than that of a previous study that used data on both physiological and behavioral parameters obtained using an ear-attached device [15]. In the study, calving within the 24 h and 6 h periods were predicted with a sensitivity of 36% and 49% and a precision of 60% and 11%, respectively, based on ear surface temperature, activity level, rumination time, feeding time, and days to the expected calving date [15]. The differences between values in the present study and the previous literature might be ascribed to the temperature measurement site, and the behavioral parameters used to develop the prediction models. Although the values were reported separately from two different study groups, the Pearson’s correlation coefficients between ST and core body temperature (vaginal temperature) (0.56) [34] tended to be higher than that between ear surface temperature and core body temperature (rectal temperature) (0.22) [35]. This implies that ST reflects the core body temperature more precisely than the ear surface temperature. Furthermore, the present behavioral parameters (lying time, posture change, and tail raising) may be more directly related to calving than rumination and eating behavior, judging by their calving prediction efficacy [36,37,38].

Although the optimal time window is debatable, we developed calving prediction models to predict 24-h and 6-h periods before calving. These time windows were chosen based on the recommended calving management [16]; moving cattle to the maternity pen once signs of the first stage of calving have been detected (12 to 24 h before calving) and detecting the onset of the second stage of calving (the interval from the appearance of the allantoic/amniotic sac to the passage of the fetus, which takes 0.5 to 4 h [39]) to detect abnormal calving early and to give timely intervention. Since the present mean intervals from 24-h and 6-h calving alerts to actual calving (12.0 h and 2.5 h, respectively) fall within the ranges, the 24-h and 6-h calving alerts would be helpful to implement timely observation to detect signs of the first and second stages of calving, respectively. Therefore, the present two-stage calving prediction alerts may be useful for ensuring proper calving management.

A biphasic decrease in ST before calving was observed in this study, which is con-sistent with previous studies using a comparable tail-attached device [10,40]. In our previous study, calving within the 24 h period was predicted with a sensitivity of 84% and a precision of 71% based on the continuous measurements of ST and days to the expected calving date [10]. The relatively higher predictive ability of the present models than the previous study indicates that behavioral data have additional value for a more accurate prediction of calving, as hypothesized. This might also be supported by the present results of feature importance analysis; several behavioral parameters-related features were regarded as important. However, a follow-up study is needed to improve calving prediction models, especially precision, since farmers may prefer the fewest false positive alerts possible [15].

In untethered cattle, the present changes in activity intensity, lying time, posture change, and tail raising around calving correspond with the findings of previous studies. Before calving, activity intensity (or the number of steps) increased in the last 6–12 h [23,24,41], lying time decreased in the last 24 h [24,25], lying bouts increased in the last 6–12 h [22,23,24], and tail raising increased in the last 2–6 h [22,25]. The lack of a decrease in lying time and relatively earlier onset of increased posture changes in tethered cattle may indicate frustration resulting from interference with prepartum isolation-seeking behavior, which is observed within 24 h before calving under untethering condition [42]. This might be supported by the present similar pre-calving sequential changes in the posture change ratio under the tethering condition and the activity intensity ratio under the untethering condition.

Interestingly, calving prediction models developed under tethering and untethering conditions showed similar predictive performance, regardless of the differences in pre-calving behavioral changes under these two housing conditions. Furthermore, the mean intervals from alerts to actual calving of the models were also similar. These results indicate that machine learning prediction models based on the data on physiological and behavioral parameters obtained by a tail sensor and days to the expected calving date have the potential to achieve effective calving prediction, irrespective of the housing conditions in cattle.

## 5. Conclusions

We developed calving prediction models for 24-h and 6-h periods before calving using data on physiological and behavioral parameters obtained using a multimodal tail-attached device (tail sensor) together with a non-sensor-based data “days to the expected calving date” under two distinct housing conditions: tethering and untethering. LOOA cross-validation showed that calving within the following 24 h period under tethering and untethering conditions was predicted with a sensitivity of 97% and 93% and precision of 80% and 76%, respectively, while calving within the 6 h period was predicted with a sensitivity of 91% and 90% and precision of 88% and 90%, respectively. The results of the current study indicate that calving prediction models based on data on physiological and behavioral parameters obtained by a tail sensor with supervised machine learning have the potential to achieve effective calving prediction, irrespective of the housing conditions in cattle.

## 6. Patents

Drs. Higaki and Yoshioka have patents pending for the use of the tail-attached device for calving prediction and estrus detection (Japanese Patent Pending No. 2019-152271) and the device attachment (Japanese Patent Pending No. 2021-29164).

## Figures and Tables

**Table 1:** Description of features used to build the calving prediction models for predicting 24-h and 6-h periods before calving through supervised machine learning.

Feature Description
Features derived from sensor data
Smoothened rST and ratios of activity intensity, lying time, and posture change
Minimum value during the last 12 h (6 h) *
Maximum value during the last 12 h (6 h)
Minimum value during the last 24 h (12 h)
Maximum value during the last 24 h (12 h)
Difference between the current value and minimum value during the last 12 h (6 h)
Difference between the current value and maximum value during the last 12 h (6 h)
Difference between the current value and minimum value during the last 24 h (12 h)
Difference between the current value and maximum value during the last 24 h (12 h)
Ratio of tail raising index
Minimum value during the last 6 h (3 h)
Maximum value during the last 6 h (3 h)
Minimum value during the last 12 h (6 h)
Maximum value during the last 12 h (6 h)
Difference between the current value and minimum value during the last 6 h (3 h)
Difference between the current value and maximum value during the last 6 h (3 h)
Difference between the current value and minimum value during the last 12 h (6 h)
Difference between the current value and maximum value during the last 12 h (6 h)
Feature derived from non-sensor-based data
Days to the expected calving date

The residual tail skin temperature (rST) was calculated as the actual ST − mean ST for the same time on the previous three days. The rST data were smoothened using the exponentially weighted moving average and used for feature extraction. Ratios of activity intensity and lying time were calculated as the total value during the last 24 h/total value during the last 25–48 h. The ratio of posture change was calculated as the average number during the last 6 h/average number during the last 7–30 h. The ratio of tail raising index was calculated as the average value during the last 3 h/average value during the last 4–27 h. * Times in parentheses are used to extract the features for developing the 6-h prediction models.

**Table 2:** Performance of the calving prediction models for predicting 24-h and 6-h periods before calving under tethering and untethering conditions.

Housing Condition	Prediction Period	True Positive	False Negative	False Positive	Sensitivity (%) ^1^	Precision (%) ^2^	Average Time Interval (h) ^3^
Tethering							
(n = 33)	24 h	32	1	8	97.0	80.0	13.7 ± 1.0
	6 h	30	3	4	90.9	88.2	2.7 ± 0.4
Untethering							
(n = 30)	24 h	28	2	9	93.3	75.7	10.2 ± 1.5
	6 h	27	3	3	90.0	90.0	2.3 ± 0.3
Total							
(n = 63)	24 h	60	3	17	95.2	77.9	12.0 ± 0.9
	6 h	57	6	7	90.5	89.1	2.5 ± 0.3

^1^ Sensitivity was calculated as true positive/(true positive + false negative). The values of the calving prediction models developed under tethering and untethering conditions were not significantly different under the corresponding prediction periods (*p* > 0.05, Fisher’s exact test). ^2^ Precision was calculated as true positive/(true positive + false positive). The values of the calving prediction models developed under tethering and untethering conditions were not significantly different under the corresponding prediction periods (*p* > 0.05, generalized score statistic). ^3^ Average time intervals (mean ± standard error) from true-positive calving alerts to actual calving. The values of the calving prediction models developed under tethering and untethering conditions were not significantly different under the corresponding prediction periods (*p* > 0.05, two-tailed Student’s *t*-test).

## Data Availability

The datasets generated during and/or analysed during the current study are available from the corresponding author on reasonable request.
